# Cure of Anal Fissure without Operation

**Published:** 1903-09

**Authors:** 


### ABSTRACT.

---

### Cure of Anal Fissure Without Operation.

S. Lewis, of Brooklyn, (*Medical News,* May 30, 1903,) reports eight cases and says the following regarding the after-treatment:

In contradiction to one of the leading authorities (Gant, Dis. of Rectum and Anus, p. 124), I am amazed to find the "severe pain and tenesmus" following the use of suppositories absent in my cases. I ascribe this: (1) to the special action of the permanganate and, (2) the satisfactory shape and consistency of the suppository employed, and care in its use. I have discarded a former favorite (a combination of Peru balsam and iodoform) and employ a suppository sold under the name of anusol. This affords an absolute uniformity of shape and consistency and presents a more astringent and stimulating action on the fissure and on hemorrhoids, if present. Best of all, it pulpefies the stool, which is allowed to be passed daily, a much simplier method than that of Boas, who stops the movements for a week or more. In my worse cases I have inserted the suppository myself or instructed a competent nurse. It should be employed night and morning, warmed till slippery, then gently pressed against the anal orifice on the side opposite the fissure until the sphincter relaxes and the suppository slips in. In most cases it can be inserted by the patient. I cannot feel that ointments, as recommended by Gant, or powders (Boas), could be relied on to reach the involved area if applied by the patient. One suppository is ordered night and morning, the usual dietetic and medicinal treatment of constipation prescribed, and patient told to report in two days. Severe cases are told to rest as much as possible, reclining. The worst are confined to bed.

In every one of my cases the next stool has been loose, mixed with products of the melted suppository and marked by two or three minutes of smarting in cases in which for days and weeks, even months, preceding the patients have after every stool rolled in agony, lasting sometimes two hours.

Condition at next treatment: spasm and inflammation greatly lessened, ulcer covered by clean granulating area. Treatment as before, but cocain omitted, patients to report in four days, or sooner, if any pain exists. At third treatment, suppositories may be reduced to one daily, at bed-time.

---

HEMATEMESIS FOLLOWING APPENDICECTOMY.

George Ryerson Fowler, (*Brooklyn Med. Jour.*, July, 1903,) reports a fatal case. The occurrence of gastric hemorrhage has added another to the already long list of grave sequelæ of appendicitis. Whether this hemorrhage depends upon the toxic effects of septic bacterial products which enter the circulation and produce necrosis of the cells of the gastric mucosa, this in its turn being followed by ulceration due to the digestive action of the gastric juice, as claimed by Nitsche, or upon embolism of the vessels of the gastric mucosa, as shown in the case herewith reported, matters but little from the clinical standpoint. In either case the toxicity of the appendical lesion is responsible for the fatal issue which in the vast majority of cases ensues.

